# Both male and female obese ZSF1 rats develop cardiac dysfunction in obesity-induced heart failure with preserved ejection fraction

**DOI:** 10.1371/journal.pone.0232399

**Published:** 2020-05-06

**Authors:** Isabel T. N. Nguyen, Maarten M. Brandt, Jens van de Wouw, Ruben W. A. van Drie, Marian Wesseling, Maarten J. Cramer, Saskia C. A. de Jager, Daphne Merkus, Dirk J. Duncker, Caroline Cheng, Jaap. A. Joles, Marianne C. Verhaar

**Affiliations:** 1 Department of Nephrology and Hypertension, University Medical Center Utrecht, Utrecht, The Netherlands; 2 Division of Experimental Cardiology, Department of Cardiology, Erasmus MC, University Medical Center Rotterdam, Rotterdam, The Netherlands; 3 Laboratory of Experimental Cardiology, University Medical Center Utrecht, Utrecht University, Utrecht, The Netherlands; 4 Department of Cardiology, University Medical Center Utrecht, Utrecht, The Netherlands; 5 Walter Brendel Center of Experimental Medicine (WBex), Munich, Germany; 6 German Center for Cardiovascular Research (DZHK), Partner Site Munich, Munich Heart Alliance (MHA), Munich, Germany; Scuola Superiore Sant’Anna, ITALY

## Abstract

Heart failure with a preserved ejection fraction (HFpEF) is associated with multiple comorbidities, such as old age, hypertension, type 2 diabetes and obesity and is more prevalent in females. Although the male obese ZSF1 rat has been proposed as a suitable model to study the development of diastolic dysfunction and early HFpEF, studies in female animals have not been performed yet. Therefore, we aimed to characterize the cardiac phenotype in female obese ZSF1 rats and their lean counterparts. Additionally, we aimed to investigate whether differences exist in disease progression in obese male and female ZSF1 rats. Therefore, male and female ZSF1 rats, lean as well as obese (N = 6-9/subgroup), were used. Every two weeks, from 12 to 26 weeks of age, systolic blood pressure and echocardiographic measurements were performed, and venous blood was sampled. Female obese ZSF1 rats, as compared to female lean ZSF1 rats, developed diastolic dysfunction with cardiac hypertrophy and fibrosis in the presence of severe dyslipidemia, increased plasma growth differentiation factor 15 and mild hypertension, and preservation of systolic function. Although obese female ZSF1 rats did not develop hyperglycemia, their diastolic dysfunction was as severe as in the obese males. Taken together, the results from the present study suggest that the female obese ZSF1 rat is a relevant animal model for HFpEF with multiple comorbidities, suitable for investigating novel therapeutic interventions.

## Introduction

Heart failure (HF) is a growing global health problem, with high morbidity and mortality, and the leading cause for hospitalizations in patients above 65 years of age [1]. HF can be divided into three types: HF with a preserved ejection fraction (HFpEF), HF with mid-range ejection fraction (HFmrEF) and HF with reduced ejection fraction (HFrEF). HFpEF is characterized by an increase in left ventricular (LV) wall thickness and/or left atrial size as indication of increased LV filling pressures. Additionally, impaired LV filling, also referred to as diastolic dysfunction, is observed in these patients [2]. Although diastolic dysfunction is highly prevalent in HFpEF, the disease is viewed as a complex syndrome in which cardiac and non-cardiac determinants contribute to the impairment of the cardiovascular reserve [3, 4]. Conversely, HFrEF is defined by a lack of contractility with ejection fraction (EF) <40%, caused by an insult that leads to myocyte loss and functional impairment [5]. HFmrEF is the subgroup of patients with symptoms of HF and an EF ranging from 40 to 49%. Although effective treatment options exist for HFrEF and HFmrEF, this is not true for HFpEF [6]. Its underlying mechanisms are poorly understood and are further complicated by the presence of multiple interrelated comorbidities such as obesity, diabetes mellitus type 2 and hypertension [7, 8]. HFpEF is more predominant in elderly post-menopausal women and is closely associated with the presence of hypertension as well as excess weight or obesity [9, 10]. To better understand the underlying mechanisms and the effects of new therapies, animal models of early HFpEF are required. As it is not possible to mimic every detail of the disease in an animal model, the focus should be on specific pathophysiological aspects of early HFpEF in patients [11, 12].

In this regard, the male obese Zucker diabetic fatty/Spontaneously hypertensive heart failure F1 hybrid (ZSF1) rats have been proposed as an animal model for HFpEF [13, 14]. This model develops by crossing two rat strains with two different leptin mutations (*fa* and facp): the lean female Zucker diabetic fatty rat (ZDF; +/fa) and the lean male spontaneously hypertensive heart failure rat (SHHF; +/facp) [15]. Previous studies have shown that male obese ZSF1 rats develop diastolic dysfunction between 10 and 20 weeks of natural aging with concentric LV remodeling and hypertrophy [16].

To date, all studies using this model to mimic human HFpEF disease have been performed in male animals, while clinical prevalence of HFpEF is higher in women. In non-obese rat strains, male rats are more prone to develop HF compared to females and often the phenotype in females is milder compared to their male counterparts [17, 18]. As a result, female animals are often not included in experimental studies. Consequently, we aimed to characterize the cardiac phenotype in female obese ZSF1 rats and their lean counterparts in the current study. Additionally, it is not clear whether the disease progression is more severe in women compared to men. Therefore, we also aimed to investigate whether differences exist in disease progression in obese male and female ZSF1 rats. Our findings demonstrate that female obese ZSF1 rats develop diastolic dysfunction compared to lean female ZSF1 rats and that severity and progression of the disease within the timeframe of the study were similar between male and female obese ZSF1 rats. This indicates that female obese ZSF1 rats could be considered a representative model for interventional studies on diastolic dysfunction.

## Materials and methods

### Animals

All procedures were approved by the Animal Ethics Committee of the University of Utrecht (CCD: AVD115002016462) and were in accordance with the Dutch Codes of Practice for the Care and Use of Animals for Scientific Purposes. Experiments were conducted in nine-week old male lean (N = 8), male obese (N = 9), female lean (N = 6) and female obese (N = 8) ZSF rats obtained from Charles River (Kingston, MA, USA) that were co-housed in a climate-controlled facility with a 12-hour light/dark cycle. As the obese rats contain two different leptin mutations (*fa* and facp), they become hyperphagic and develop obesity naturally. Rats had access to water and standard rat chow (CRM-E; Special Diet Services, Witham, Essex, UK) ad libitum. From 12 weeks to 26 weeks of age, as described previously [19], systolic blood pressure was measured every two weeks via tail-cuff plethysmography in awake rats. At 12 weeks and every two weeks from 18 to 26 weeks of age echocardiography was performed and blood sampled from the tail vein in anesthetized rats. Body weight was monitored every week. After the last measurements, rats were administered buprenorphine (0.015 mg/kg) and were anaesthetized with isoflurane (4% for induction, 2.5% for maintenance) for terminal measurements. At the end, rats were euthanized by exsanguination via the femoral artery and organs were harvested and weighed.

### Echocardiographic evaluation

Transthoracic echocardiography was performed with a digital ultrasound machine (Sonos 5500, Philips Research, Eindhoven, the Netherlands) and a 15 MHz linear array transducer (Hewlett Packard, Palo Alto, CA). Animals were anesthetized with isoflurane (3.5–4% for induction and 2.5% for maintenance) and placed in supine position on a heating pad. Two-dimensional B-mode cine loops were recorded in the parasternal long-axis and the midpapillary short-axis views. LV volume was calculated with the area-length method at end-diastole and end-systole in the parasternal long-axis view [20]. Cardiac output and ejection fraction were then calculated from these LV volumes. Systolic and diastolic wall thickness and cavity dimensions were recorded in M-mode in the short-axis view. Images from apical 4-chamber view were acquired to evaluate LV filling and diastolic function. Mitral flow velocity tracings were obtained with pulsed-wave Doppler. Peak early E (E wave) filling velocities were measured. Tissue Doppler Imaging was used to obtain early (e’) diastolic velocity at the medial mitral annulus. For evaluation of diastolic dysfunction, the ratio of E over e’ was calculated. Volumes and dimensions were normalized for body surface area [21]. Typical study duration was 15 min. The acquisitions were coded for blinded analysis. The recordings were analyzed offline using the software present on the system and the variables were measured in at least three heart beats at end-diastole and end-systole.

### Plasma measurements

Immediately after each echocardiography session, a blood sample of 0.5 ml was drawn from the tail vein into EDTA anticoagulant-coated tubes, while the rats were still under anesthesia. Plasma was extracted by centrifugation at 3500 x g for 15 minutes at 4°C and used for determination of plasma glucose, cholesterol and triglycerides (DiaSys Diagnostic Systems GmbH, Cholesterol FS, Triglycerides FS, Glucose GOD FS, Waterbury, CT, USA). Circulating GDF15 levels were measured with a rGDF15 ELISA (MGD150, Quantikine ELISA, R&D systems Inc., Minneapolis, MN). Before use, plasma samples were prediluted 1:50 with dilution buffer according to the manufacturers protocol.

### Histological analysis

Deparaffinized cardiac sections were stained with Picro Sirius Red (Sigma-Aldrich, Zwijndrecht, the Netherlands) for 25 min, differentiated in 0.2 HCl and rinsed with aquadest, followed by dehydration. Of each section, 20 non-overlapping fields were imaged with a fluorescence microscope with a polarizing filter (Olympus BX51, magnification 100x). Analysis was performed in a blinded fashion using Adobe Photoshop and ImageJ Software.

To distinguish between collagen type I and III fibers, eight fields were examined in the mid-myocardial layer of each slide and analyzed using a polarization filter (magnification 20x). Blood vessels, including perivascular collagen, tissue ruptures and folds were excluded. The birefringence capacity of the collagen fibers is used to differentiate between the thick collagen fibers (red-yellow, Type I) and the thin fibers, with a lower birefringence, (green, Type III). The area occupied by collagen type I and type III fibers was measured and expressed as percentage of the myocardial area. All measurements were performed using a microscopy image analysis system (Impak C, Clemex Vision Image analysis system, Clemex Technologies, Quebec, Canada).

Deparaffinized cardiac sections for lectin staining were subjected to 3% hydrogen peroxide in PBS for 30 min, followed by heat-induced antigen retrieval in citrate/HCl buffer (pH 6.0) for 15 min. To visualize capillaries, sections were first blocked in avidin and biotin blocking solution (Abcam). Sections were then incubated overnight at 4°C with biotin labelled anti-Lectin (1:200, Sigma-Aldrich), after which HRP-bound streptavidin (1:500, Bio-Rad Laboratories) was added to the samples for 60 min. Finally, 3,3′-diaminobenzidine solution was applied to the sections twice, 6 min each. Four non-overlapping fields in the sub-endocardium were imaged and analyzed in a blinded fashion.

Gomori staining was performed using the Reticulum Stain Kit (Diagnostic Biosystems) according to manufacturer’s instructions. Slides were counterstained for 3 min using Nuclear Fast Red and imaged. Cross-sectional areas of cardiomyocytes with clearly visible nuclei, and height to width ratios not exceeding 1:2, were measured in 4 non-overlapping fields in a blinded fashion using Clemex software.

### Statistics

Data are presented as mean ± SEM. Groups were compared by 2-way ANOVA for repeated measurements followed by Bonferroni post hoc test using GraphPad Prism 7.0 software (GraphPad, San Diego, CA). Pre-terminal and terminal variables at 26 weeks of age were compared by 2-way ANOVA followed by Tukey multiple comparison test. P<0.05 was considered significant.

## Results

### Metabolic profile

**Fig 1:**
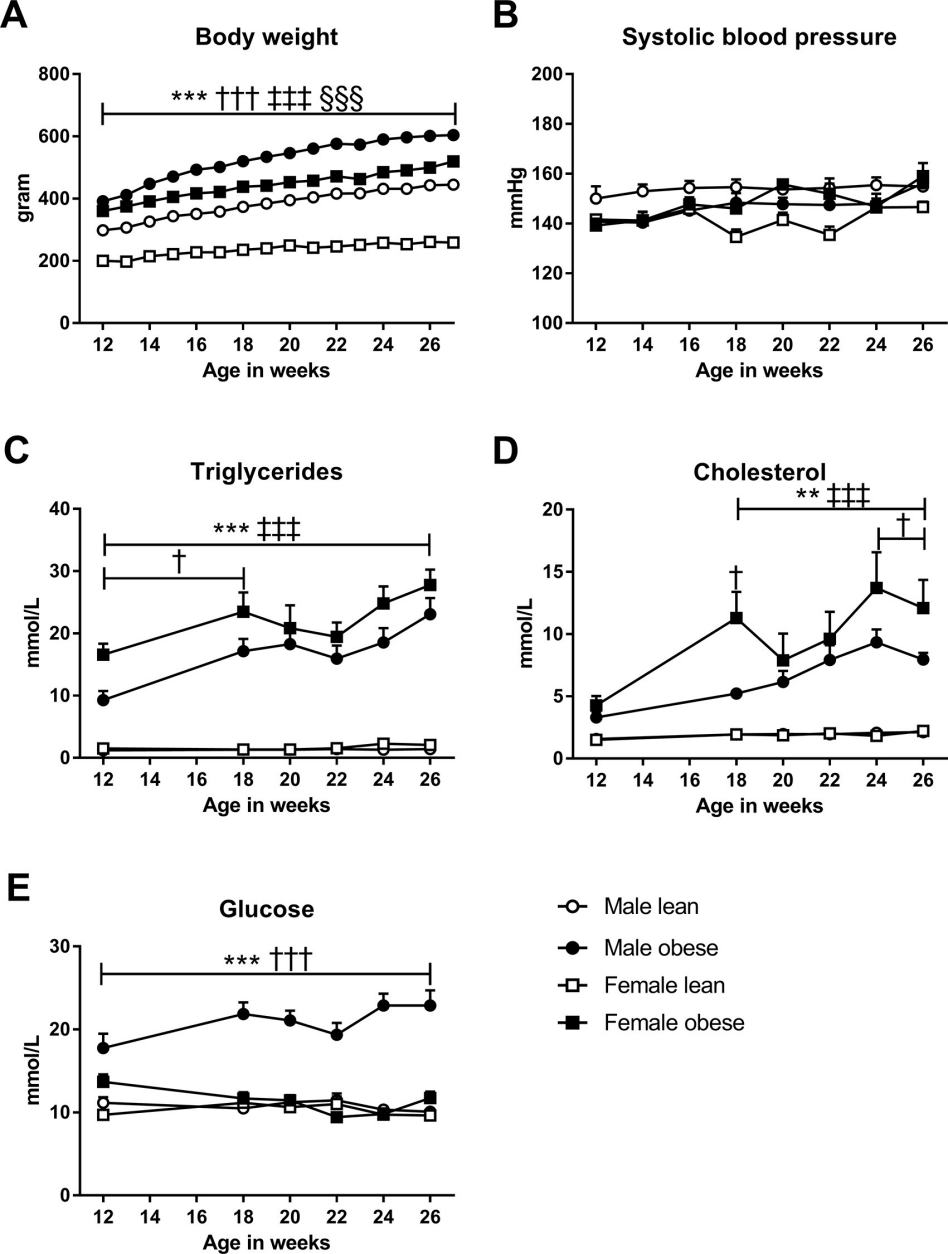
Both male and female obese ZSF1 rats exhibit obesity, dyslipidemia and mild hypertension. **A)** Body weight was markedly higher in the obese ZSF1 rats compared to their lean counterparts. **B)** Systolic blood pressure was similar in all the groups, with a range of 140–150 mmHg over time. **C)** Triglycerides and **D)** cholesterol levels were markedly elevated in both male and female obese ZSF1 rats. **E)** Plasma glucose levels were only elevated in male obese rats compared to their lean counterparts. Means ± SEM. Male obese N = 9; female obese N = 8; male lean N = 8 and female lean N = 6; * male obese vs. male lean; † male obese vs. female obese; ‡ female obese vs. female lean; § male lean vs. female lean. One symbol P<0.05, two symbols P<0.01, three symbols P<0.001.

Obese ZSF1 rats showed a rapid weight gain on a regular chow diet. Body weight was significantly higher in both male and female obese ZSF1 rats compared to their lean counterparts (Fig 1A). Male animals were in general heavier compared to female animals. Systolic blood pressure was similar in both obese and lean ZSF1 rats, and no sex differences were observed (Fig 1B). Dyslipidemia was present in both male and female obese ZSF1 rats compared to lean ZSF1 rats (Fig 1C and 1D) and was more pronounced in female obese than in male obese rats. Surprisingly, only the obese male ZSF1 rats became hyperglycemic, with the female obese rats showing similar glucose levels as their lean littermates (Fig 1E).

### Cardiac structure and function

**Fig 2:**
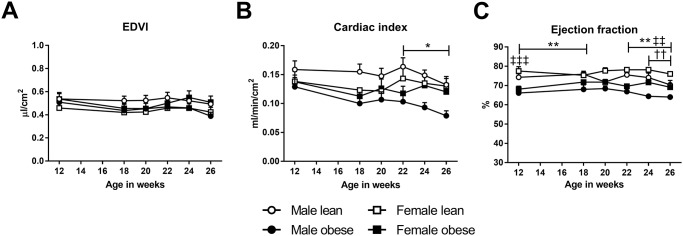
Preserved systolic function in male and female obese ZSF1 rats. **A)** End-diastolic volume index (EDVI) was not significantly different between groups. **B**) Cardiac index was decreased in obese male ZSF1 rats, while **C**) ejection fraction was preserved in all the groups. Means ± SEM. Symbols as in Fig 1.

Analysis of the left ventricle in the parasternal long-axis view showed an increased end-diastolic volume (EDV) in female obese ZSF1 rats as compared to lean female ZSF1 rats, while no differences were observed between male obese ZSF1 rats and their lean counterparts (Table 1). This increased EDV in female obese ZSF1 rats is likely due to their much larger body weight, as EDV normalized for body surface area (BSA) was similar between obese and lean females (Fig 2A). Similar patterns were found in end-systolic volume (ESV) and stroke (SV) volume, with no significant differences between obese and lean rats after normalizing for BSA. Together with the lower heart rate observed in the obese rats, cardiac index was lower in obese males compared to their lean counterparts (Fig 2B).

However, ejection fraction was not different between obese and lean animals during the whole experimental period (Fig 2C).

**Table 1:** Terminal data at 26 weeks of age, expressed as absolute values and per body surface area.

	Female lean (n = 6)	Female obese (n = 8)	Male Lean (n = 8)	Male obese (n = 9)	p-values		
					Obesity	Sex	Interaction
Body weight, g	258 ± 2	519 ± 9^‡‡‡^	445 ± 7^§§§^	604 ± 15***^†††^	<0.001	<0.001	<0.001
BSA, cm^2^	49 ± 0.4	77.5 ± 1^‡‡‡^	71.3 ± 0.8^§§§^	88.1 ± 1.5***^†††^	<0.001	<0.001	<0.001
Heart rate, bpm	397 ± 10.6	336 ± 9.9^‡‡‡^	384 ± 6.7	308 ± 6.5***	<0.001	0.02	0.39
EDV, μl	170 ± 16	314 ± 33^‡‡^	280 ± 22^§^	273 ± 21	0.011	0.17	0.006
EDV, μl/cm^2^	0.42 ± 0.04	0.51 ± 0.05	0.49 ± 0.04	0.39 ± 0.03	0.83	0.55	0.03
ESV, μl	38 ± 3	95 ± 12^‡‡‡^	85 ± 8^§§^	96 ± 4	<0.001	0.007	0.008
ESV, μl/cm^2^	0.10 ± 0.001	0.15 ± 0.02	0.14 ± 0.02	0.14 ± 0.001	0.11	0.35	0.04
SV, μl	130 ± 13	218 ± 23^‡^	199 ± 17	176 ± 17	0.095	0.48	0.006
SV, μl/cm^2^	0.32 ± 0.03	0.35 ± 0.04	0.35 ± 0.03	0.25 ± 0.03	0.35	0.24	0.06
CO, ml/min	51.9 ± 6.5	75.0 ± 9.2	76.0 ± 6.2	54.6 ± 5.5	0.91	0.80	0.004
CO, ml/min/cm^2^	0.13 ± 0.02	0.12 ± 0.02	0.13 ± 0.01	0.08 ± 0.01*	0.02	0.15	0.10
LVIDd, mm	7.0 ± 0.4	8.4 ± 0.4^‡^	8.2 ± 0.2	8.4 ± 0.3	0.019	0.10	0.077
LVIDd, μm/cm^2^	17.4 ± 1.1	13.6 ± 0.6^‡‡^	14.3 ± 0.4^§^	12.0 ± 0.5*	<0.001	0.001	0.26
LVPWd, mm	1.7 ± 0.07	1.9 ± 0.1	1.5 ± 0.1	2.1 ± 0.07**	<0.001	0.84	0.09
LVPWd, μm/cm^2^	4.2 ± 0.2	3.1 ± 0.2^‡‡‡^	2.7 ± 0.2^§§§^	3.0 ± 0.1	0.03	<0.001	<0.001
IVSd, mm	1.5 ± 0.08	1.7 ± 0.09	1.6 ± 0.1	1.8 ± 0.06	0.024	0.19	0.59
IVSd, μm/cm^2^	3.8 ± 0.2	2.7 ± 0.2^‡‡‡^	2.8 ± 0.2^§§^	2.7 ± 0.1	<0.001	0.003	0.006
HW (LV+RV), mg	876 ± 27.4	1481 ± 52.2^‡‡‡^	1357 ± 28.8^§§§^	1466 ± 26.6	<0.001	<0.001	<0.001
HW mg/cm^2^	2.2 ± 0.05	2.4 ± 0.07	2.4 ± 0.05	2.1 ± 0.05**^††^	0.52	0.39	<0.001
LV mass, mg	762 ± 65	1104 ± 66^‡‡^	905 ± 26	1250 ± 70**	<0.001	0.025	0.97
LV mass, mg/cm^2^	1.9 ± 0.2	1.8 ± 0.1	1.6 ± 0.06	1.8 ± 0.1	0.7	0.17	0.15

Values are means ± SEM. BSA = body surface area, EDV = end-diastolic volume, ESV = end-systolic volume, SV = stroke volume, CO = cardiac output, LVIDd = left ventricular internal diameter in diastole, LVPWd = left ventricular posterior wall in diastole, IVSd = interventricular septum in diastole, HW = heart weight, LV = left ventricle, RV = right ventricle. Male obese N = 9; female obese N = 8; male lean N = 8 and female lean N = 6.

* male obese vs. male lean;

^†^ male obese vs. female obese;

^‡^ female obese vs. female lean;

^§^ male lean vs. female lean.

One symbol P<0.05, two symbols P<0.01, three symbols P<0.001.

**Fig 3:**
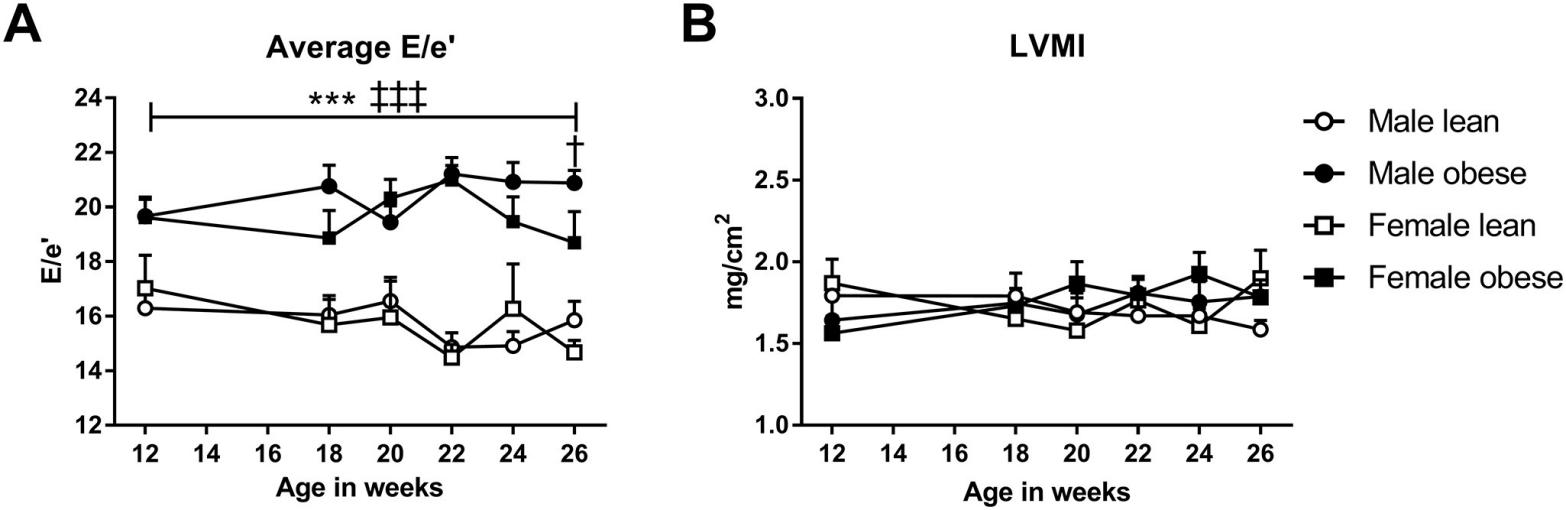
Obese ZSF1 rats show indication of diastolic dysfunction. **A)** Tissue Doppler Imaging showed that, already at an early age, the E/e’ ratio was markedly increased in both male and female obese ZSF1 rats compared to lean rats. **B)** Left ventricular mass index (LVMI) was similar between the four groups. Means ± SEM. Symbols as in Fig 1.

Tissue Doppler Imaging analysis showed that the E/e’ ratio was significantly increased in obese ZSF1 compared to lean ZSF1 rats throughout the study: from 12 to 26 weeks of age (Fig 3A). No significant differences were observed in the severity of diastolic dysfunction between obese male and female animals. When compared to their lean counterparts, both male and female obese ZSF1 rats showed higher LV mass, as calculated with echocardiography. No differences were observed between obese males and females. These echocardiography measurements of LV mass were confirmed at autopsy, with obese animals showing a higher heart weight at the terminal measurements compared to lean animals (Table 1). When normalizing these data for BSA, no significant differences were found (Fig 3B).

### Cardiac histology

**Fig 4:**
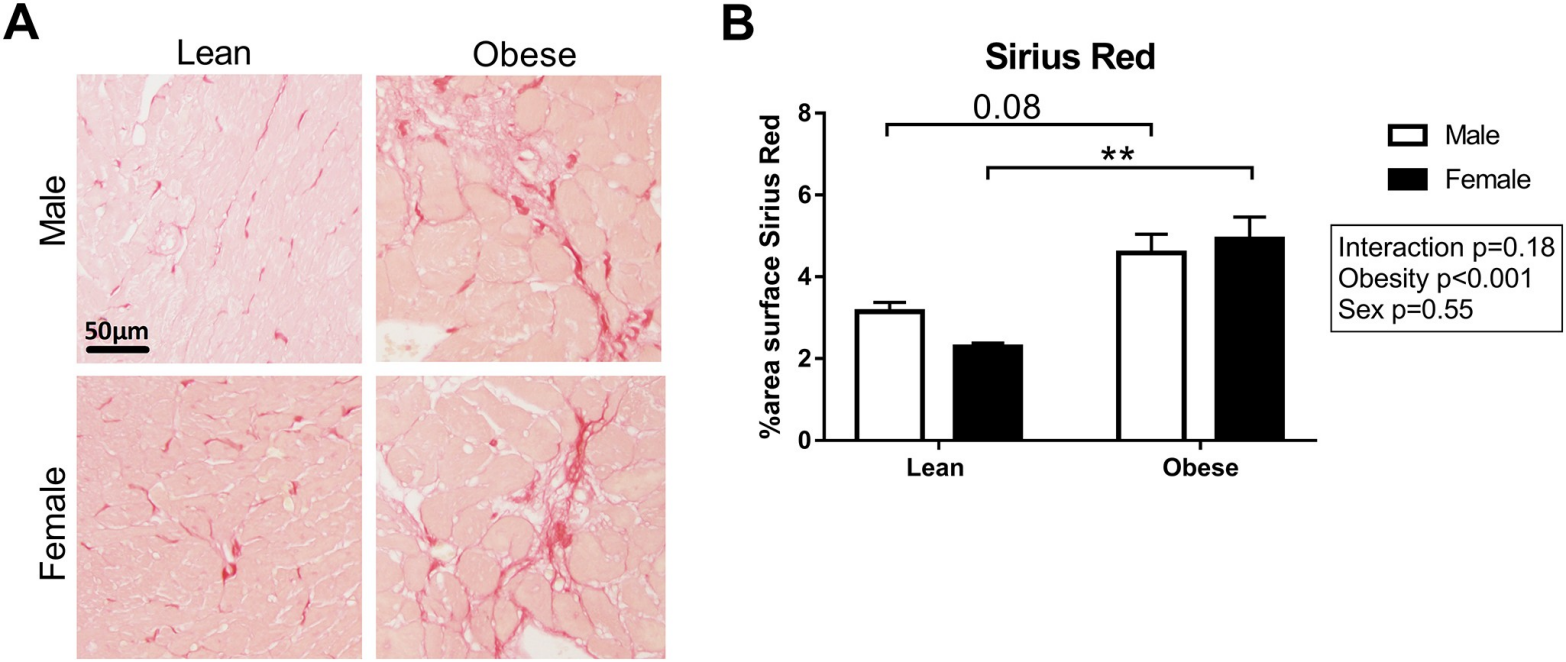
Increased fibrosis observed in obese ZSF1 rats. **A**) Representative images and **B**) quantification of histological staining of cardiac collagen fibers stained with Picro Sirius Red. N = 4-7/group. **P<0.01.

**Fig 5:**
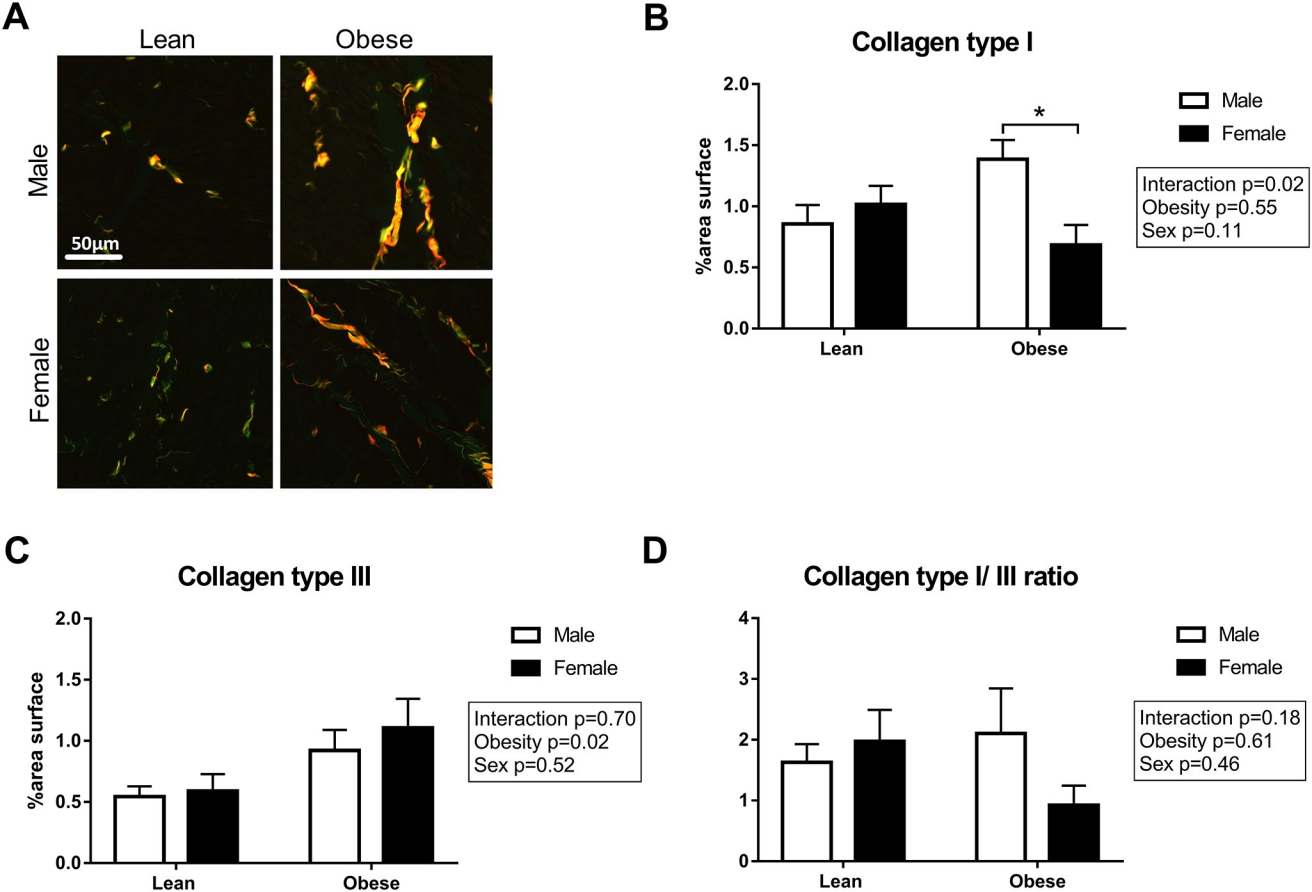
Obesity leads to an increase in collagen type III fibres. **A**) Representative images and the percentage of **B**) collagen type I fibres and **C**) type III fibres per myocardial area. **D**) Additionally, the collagen type I to type III fibre ratio was calculated in ZSF1 rats. N = 4-7/group. *P<0.05.

**Fig 6:**
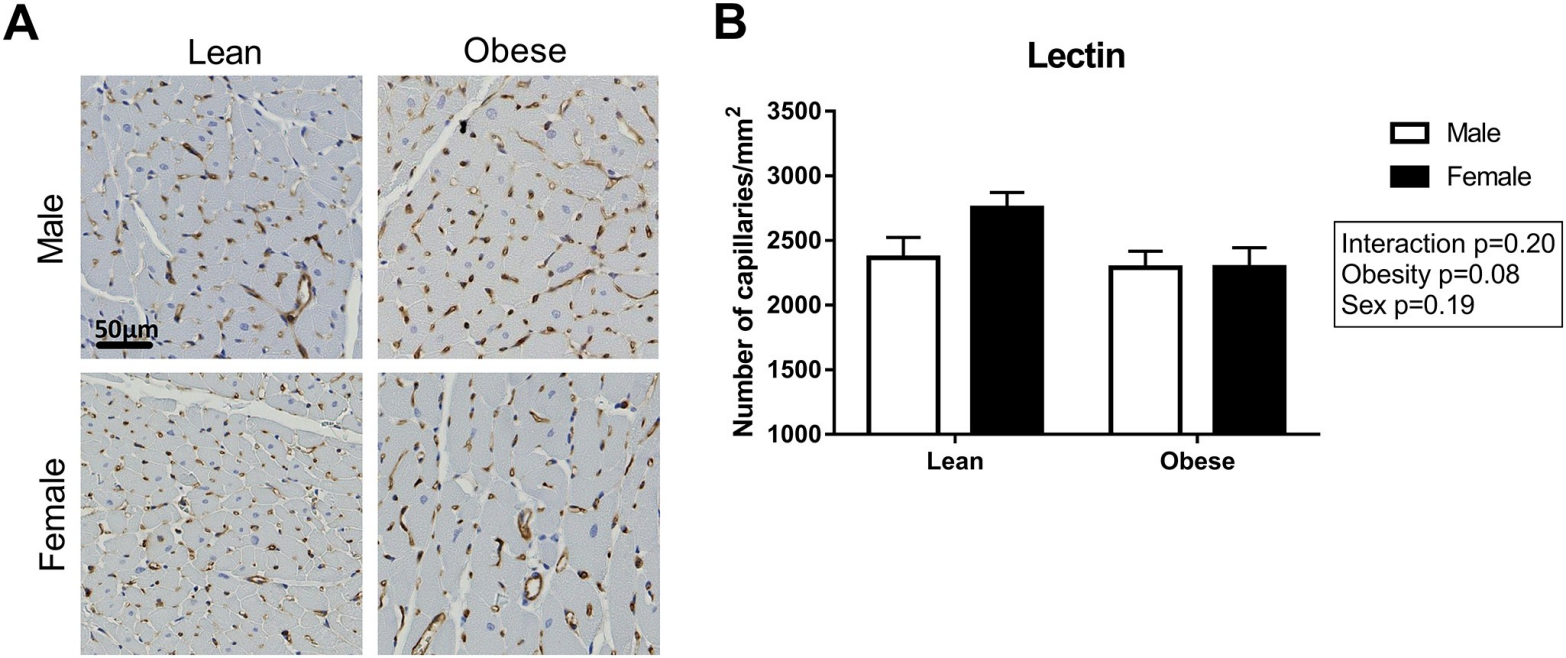
No microvascular changes were found in the ZSF1 rats. **A**) Representative images and **B**) quantification of the microvasculature stained with Lectin. N = 4-7/group.

**Fig 7:**
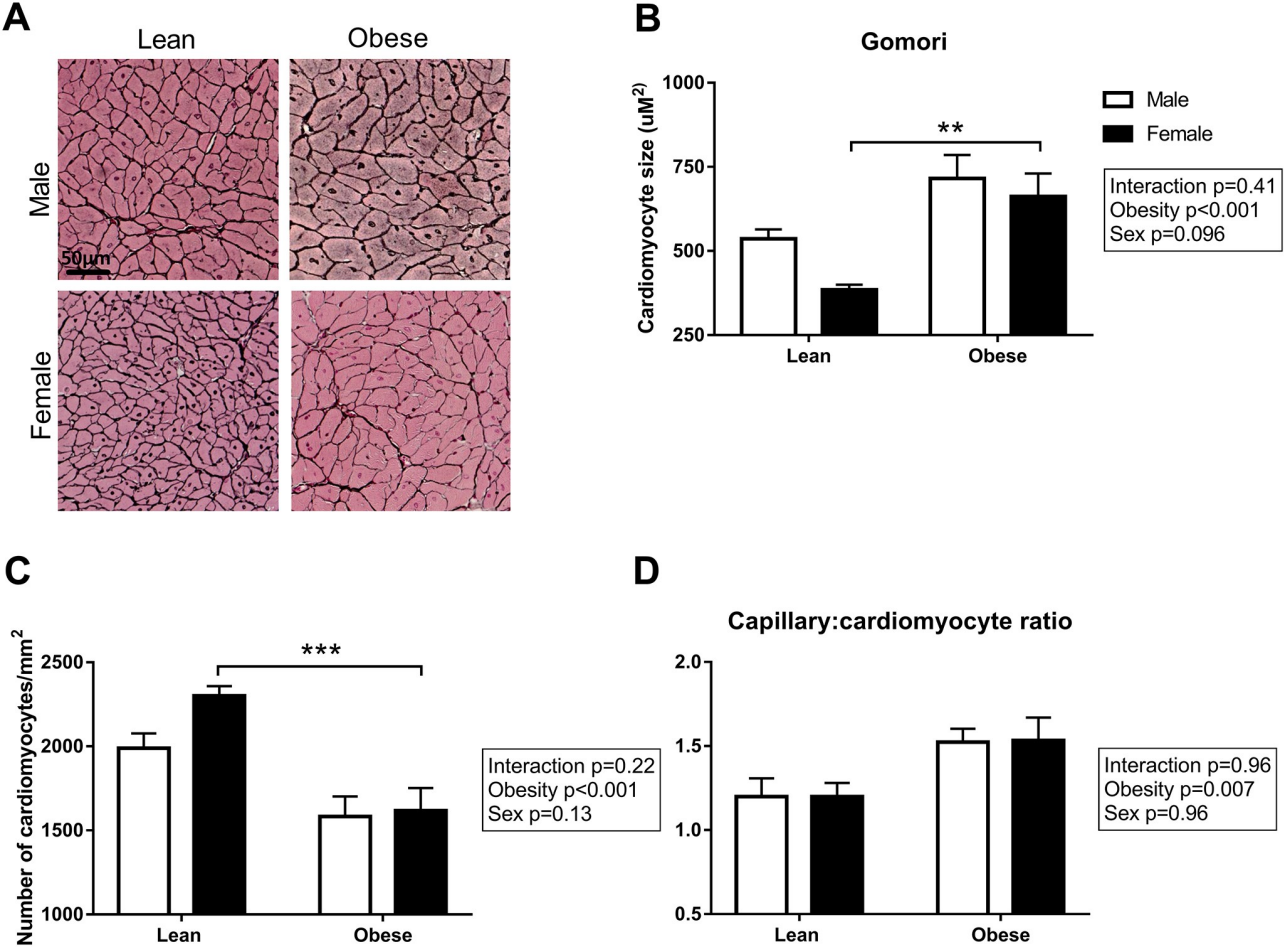
Obese female ZSF1 rats showed increased cardiomyocyte size. **A**) Representative images and **B**) quantification of cardiac hypertrophy stained with Gomori. **C**) Number of cardiomyocytes, **D**) capillary-to-cardiomyocyte ratio. N = 4-7/group. **P<0.01, ***P<0.001.

To visualize the presence of interstitial fibrosis, collagen in the heart was stained with Picro Sirius Red (Fig 4A). Interstitial fibrosis was significantly higher in obese as compared to lean rats (P<0.001; Fig 4B). There was no difference in Picro Sirius Red stained area between male and female ZSF1 rats. Additionally, the content of collagen type I and type III fibers was quantified in the LV sections (Fig 5A). Collagen type I fibers were increased in obese male ZSF1 rats compared to obese females, while collagen type III fibers were significantly higher in obese rats compared to lean rats (Fig 5B and 5C). No differences were observed in the collagen type I to type III ratio in the ZSF1 rats (Fig 5D). Capillary density was evaluated in the ZSF1 rats by performing a lectin staining on LV cross sections (Fig 6A). Surprisingly, no differences were observed between the four groups, although a trend towards a reduction in capillary density was found in obese vs. lean animals (P = 0.08; Fig 6B). Consistent with the global LV hypertrophy, Gomori staining (Fig 7A) showed cardiomyocyte-hypertrophy and a reduced number of cardiomyocytes per area in obese as compared to lean rats (P<0.001; Fig 7B and 7C). Capillary to cardiomyocyte ratio was higher in the obese animals, although no differences were observed between male and female rats (Fig 7D).

**Fig 8:**
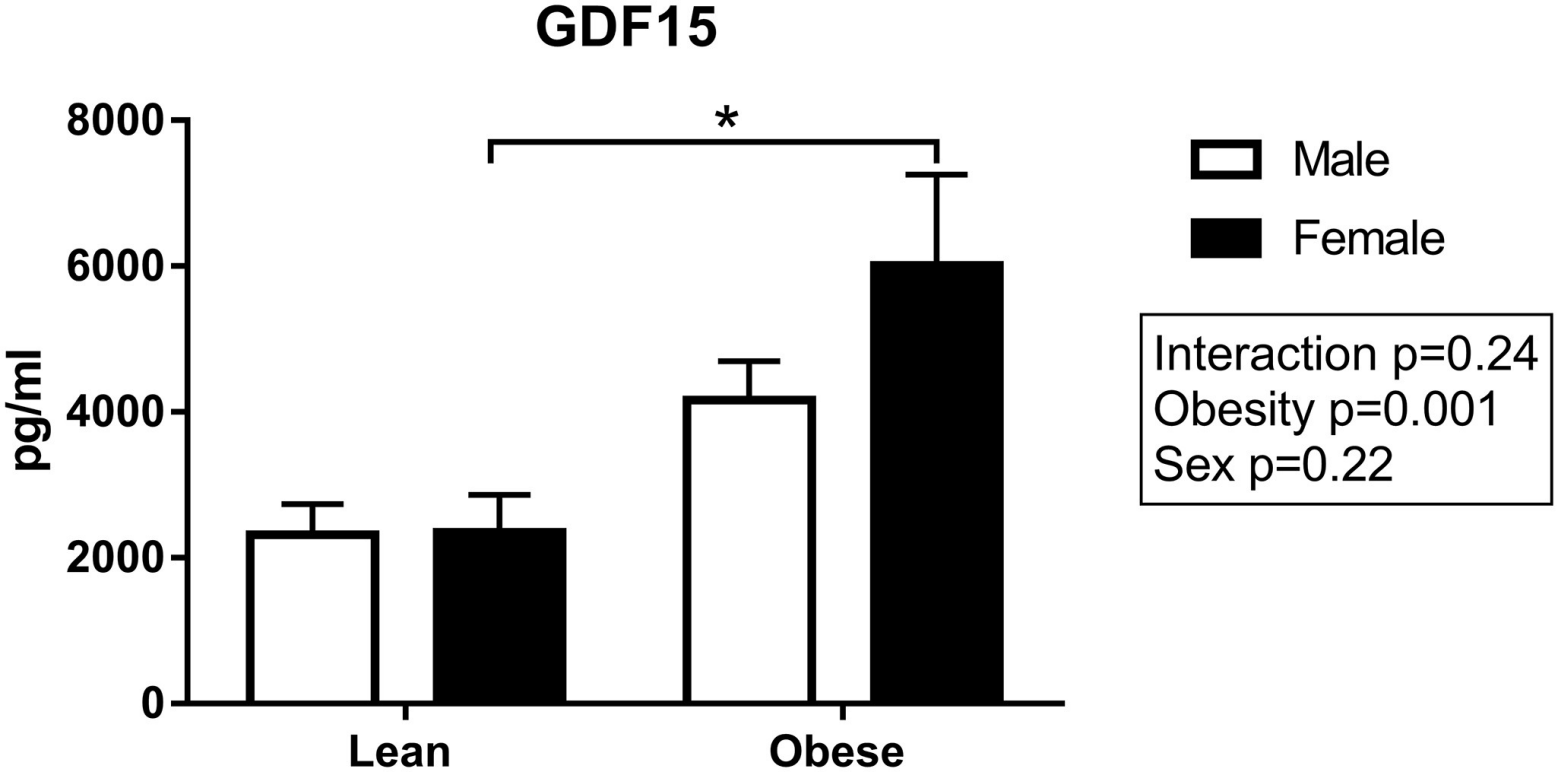
Higher levels of growth differentiation factor 15 (GDF15) in obese ZSF1 rats. GDF15 levels were measured in plasma collected at the end of the protocol. Means ± SEM. Male obese N = 9; female obese N = 7; male lean N = 7 and female lean N = 6. *P<0.05.

To further investigate the disease progression in the ZSF1 rats, the levels of growth differentiation factor 15 (GDF15) in plasma as a marker for HF were measured. A significant effect of obesity was found, with obese ZSF1 rats showing higher GDF15 levels compared to their lean counterparts (P = 0.001; Fig 8).

## Discussion

In the current study we investigated the development of early HFpEF in the female obese ZSF1 rat, a model characterized by the presence of severe obesity, dyslipidemia and mild hypertension, and compared the longitudinal disease progression between male and female obese ZSF1 rats. Obesity in the ZSF1 rats was associated with increased LV mass and diastolic dysfunction while ejection fraction was preserved. Even though female obese ZSF1 rats did not develop hyperglycemia, the absence of this risk factor did not seem to matter as severity of diastolic dysfunction was comparable between male and female obese ZSF1 rats. Our findings indicate that obese males and females both develop diastolic dysfunction.

### Contribution of metabolic syndrome to the development of diastolic dysfunction

Metabolic derangements such as obesity, hypertension, hyperglycemia and dyslipidemia are recognized as important risk factors for the development of diastolic dysfunction and HFpEF in humans [13, 22]. Obesity was observed in the ZSF1 rats, with an increase of 35% and 92% in body weight at 26 weeks of age compared to lean males and females, respectively. Hypertriglyceridemia (>16 fold in males and >17 fold in females) and hypercholesteremia (>3 fold in males and >4 fold in females) were found in the obese animals. The dyslipidemia tended to be worse in female rats which is consistent with a previous study in this model [23]. Hyperlipidemia in females has been associated with estrogen levels as an ovariectomy will correct the hyperlipidemia, while estrogen administration in ovariectomized females will exacerbate hyperlipidemia [24].

In our study, only male obese rats developed hyperglycemia from early age on. This corresponds to findings in the ZDF rats, one of the parent strains of the ZSF1 rat, in which females do not become diabetic when fed regular chow but only when a specific diabetogenic diet is provided, such as the Purina 5008 diet or Research Diets 12468 [15, 25–27]. However, the normally recommended carbohydrate-rich Purina 5008 diet for the development of diabetes, did not induce hyperglycemia in obese female ZSF1 rats compared to their lean counterparts [23]. In obese male ZDF rats, obesity seems to be the initiating factor for the development of diabetes, as caloric restriction delayed and prevented the diabetic condition [28]. Ovarian estrogen does not seem to be protective of diabetes development in obese female ZDF rats, as an ovariectomy did not lead to overt hyperglycemia in these rats [29].

As the ZSF1 rats inherit the hypertensive gene from the SHHR parent strain [30, 31], mild hypertension is observed in all groups as the normal systolic blood pressures of commonly-used laboratory rats is 120–130 mmHg [32]. However, no further differences in systolic blood pressure were found between lean and obese ZSF1 rats, similar to findings in ZDF rats [33]. In summary, apart from hyperglycemia, important risk factors associated with the development of diastolic dysfunction and HFpEF are similarly present in both male and female ZSF1 rats.

### Systolic function is preserved in the obese ZSF1 rat

Ejection fraction was preserved in the obese ZSF1 rats during the whole study period (all above 60%). Obese female showed higher EF compared to males, which has also been reported in HFpEF patients [34]. Despite this difference, overall pump function was similar between males and females. Notably, heart rate decreased over time in both male and female obese ZSF1 rats compared to their lean littermates with the biggest decrease in obese males. Although cardiovascular depression due to the isoflurane anesthesia might be a potential explanation for the decreased heart rate, telemetric recordings in conscious male ZDF rats also demonstrated lower resting heart rates in the diabetic animals compared to non-diabetic rats [35]. It has been suggested that the sympathetic system regulating the heart rate via ß-adrenoceptors is impaired during diabetes, which might explain the more severe reduction in heart rate in obese rats with hyperglycemia [36, 37]. In ZDF rats it was reported that specifically reduced ß1-adrenoreceptor activity contributed to the lower resting heart rate, at a stage when ß-adrenoceptor responsiveness to pharmacological ß1-adrenoreceptor stimulation was increased in these rats [35]. Impaired ß-adrenoceptor activity, due to desensitized ß-adrenoceptor in response to increased sympathetic nervous system activity, is consistent with the chronotropic incompetence in patients with HFpEF but may already be present at rest in these animals because rats are sympathetically dominant [38, 39].

### Obese ZSF1 rats develop diastolic dysfunction with cardiac remodeling

Previous studies have reported impaired diastolic function with left ventricular hypertrophy in male obese ZSF1 rats, however the cardiac phenotype of their female counterparts remained unknown [13, 14, 16]. Here we showed that female obese ZSF1 rats also develop diastolic dysfunction, evidenced by an increase in E/e’, as early as 18 weeks of age. The severity in disease progression was similar in obese female and male ZSF1 rats. Although current guidelines indicate that diagnosis of HFpEF should not be based on E/e’ alone, as particularly E/e’ values in the intermediate range only modestly correlate with filling pressures [40, 41], repetitive invasive measurement of filling pressures and/or pressure-volume loops in this model is not feasible. Additional evidence for a HFpEF phenotype is however evidenced by the observation that hypertrophic cardiac remodeling was present in obese ZSF1 rats with preservation of the ejection fraction. Echocardiography derived LV mass as well as wet heart weight were increased in both male and female obese ZSF1 rats (47% and 43% in males and females, respectively). It could be argued that the increase in LV weight was due, in part, to the larger body weight of the animals. We therefore present both uncorrected and body surface area-corrected data, as the most relevant correction factor for the pathophysiology of HF in models of severe obesity, for instance in the setting of weight loss due to caloric restriction [42], is a matter of debate [43].

Interestingly, LVEDV was increased in obese females, but not in the obese males. Similar observations were found in the db/db mouse, in which a more pronounced increase in EDV was found in obese females [44]. This finding is consistent with observations in normal weight and obese male and female patients with left ventricular hypertrophy, in which only obese females showed increased EDV compared to normal weight females [45]. This sex-specific effect is not readily explained and is an interesting topic for future studies.

Obesity had a profound effect on cardiac remodeling, with increased cardiac fibrosis, collagen type III fibers and cardiomyocyte size in both male and female ZSF1 rats. Endomyocardial biopsies from patients with HFpEF revealed more extensive hypertrophy compared to HFrEF patients [46]. Additionally, obesity had a significant effect on GDF15 levels, with higher levels observed in obese rats compared to their lean counterparts. GDF15 is a distant member of the TGF-ß superfamily and has been proposed as a biomarker in HF [47]. Circulating GDF15 levels were elevated with HFpEF and were associated with an impairment in exercise capacity [48]. It has also been found that GDF15 levels were positively correlated with myocardial fibrosis and the levels of PICP and PIIINP, markers for the synthesis of collagen type I and III [49, 50].

### The female obese ZSF1 rats show indication of diastolic dysfunction

The currently available animal models have attempted to reproduce the risk factors that lead to the development of diastolic dysfunction, such as obesity, diabetes and hypertension [12]. Many of these studies have been limited to male animals, although it is well recognized that there are differences in cardiovascular disease between women and men [51]. Female rats are often excluded as their cardiac phenotype tends to be milder and more subtle compared to male rats [52]. Recently, left ventricular diastolic dysfunction was shown in female swine in the presence of hypercholesteremia, hyperglycemia and hypertension [53]. In obese mice, females developed more severe obesity, increased blood pressure and a similar degree of diastolic dysfunction compared to males [44]. In the current study we showed that female obese ZSF1 rats develop diastolic dysfunction with cardiomyocyte hypertrophy and cardiac fibrosis, in the presence of severe dyslipidemia and mild hypertension. Remarkably, despite the absence of hyperglycemia, the severity of disease progression in the obese females was equal to the male rats. This observation suggests that hyperglycemia actually has a minor contribution to the development of diastolic dysfunction in this strain. Indeed, in 45 week old ZDF rats, severe diabetes only resulted in a relatively mild impairment of diastolic function [54]. Additionally, in streptozotocin-induced diabetic female mice, diastolic dysfunction was observed despite their less pronounced hyperglycemia compared to males [55]. This supports the notion that hyperglycemia is not a critical factor in the onset of diastolic dysfunction, but that the combination of obesity and hypertension might be more important.

It could be argued that an ovariectomy in the obese female ZSF1 rats might lead to a different cardiac phenotype, as in clinical practice HFpEF is predominantly present in elderly post-menopausal women. An ovariectomy in obese Zucker females led to a decrease in plasma triglycerides [24]. Thus, in contrast to what one would expect, an alleviation in the metabolic syndrome phenotype was observed after ovariectomy and consequently also in the disease progression. Additionally, in obese female ZDF rats, ovariectomy did not lead to overt hyperglycemia [29].

In conclusion, we showed that the female obese ZSF1 rat developed diastolic dysfunction with cardiac hypertrophy and fibrosis in the presence of metabolic derangements. Their observed cardiac phenotype was as severe as obese male rats, indicating that female obese ZSF1 rats should not be excluded from future studies investigating the mechanisms underlying diastolic dysfunction and early HFpEF. We believe that this model is useful in enhancing our understanding on the contribution of metabolic factors to this cardiac disease. Additionally, this model is suitable for testing novel therapeutic interventions.

## Supporting information

S1 Dataset Raw data. (XLSX)
